# Dissemination of Ceftriaxone-Resistant *Salmonella* Enteritidis Harboring Plasmids Encoding *bla*_CTX-M-55_ or *bla*_CTX-M-14_ Gene in China

**DOI:** 10.3390/antibiotics13050456

**Published:** 2024-05-16

**Authors:** Siyuan Yang, Jianzhong Fan, Lifei Yu, Jintao He, Linghong Zhang, Yunsong Yu, Xiaoting Hua

**Affiliations:** 1 Department of Infectious Diseases, Sir Run Run Shaw Hospital, School of Medicine, Zhejiang University, Hangzhou 310016, China; 22118636@zju.edu.cn (S.Y.); yulifei@zju.edu.cn (L.Y.); jintao_he@zju.edu.cn (J.H.); 1199200152@zju.edu.cn (L.Z.); 2 Key Laboratory of Microbial Technology and Bioinformatics of Zhejiang Province, Hangzhou 310016, China; 3 Regional Medical Center for National Institute of Respiratory Diseases, Sir Run Run Shaw Hospital, School of Medicine, Zhejiang University, Hangzhou 310016, China; 4 Department of Clinical Laboratory, Affiliated Hangzhou First People’s Hospital, School of Medicine, Westlake University, Hangzhou 310006, China; assit500@163.com; 5 Department of Infectious Diseases, Affiliated Hangzhou First People’s Hospital, School of Medicine, Westlake University, Hangzhou 310006, China

**Keywords:** ceftriaxone-resistant *Salmonella* Enteritidis, CTX-M enzymes, IS*26*, IS*Ecp1*, plasmid fusion, type IV secretion system

## Abstract

*Salmonella* Enteritidis was the primary foodborne pathogen responsible for acute gastroenteritis. The growing ceftriaxone resistance poses a significant threat to public health. Infection with *S.* Enteritidis has emerged as a major public health concern, particularly in developing countries. However, research on ceftriaxone-resistant *S.* Enteritidis (CRO-RSE) remains limited, particularly concerning its resistance mechanism, plasmid structure, and transmission characteristics. This study aims to address these gaps comprehensively. We collected 235 *S.* Enteritidis isolates from Hangzhou First People’s Hospital between 2010 and 2020. Among these, 8.51% (20/235) exhibited resistance to ceftriaxone. Whole-genome analysis revealed that 20 CRO-RSE isolates harbored *bla*_CTX-M-55_ or *bla*_CTX-M-14_ on the plasmid. Moreover, the dissemination of the *bla*_CTX-M-type_ gene was associated with IS*26* and IS*Ecp1*. Plasmid fusion entailing the integration of the p1 plasmid with antibiotic resistance genes and the p2 (pSEV) virulence plasmid was observed in certain CRO-RSE. Additionally, the structural analysis of the plasmids unveiled two types carrying the *bla*_CTX-M-type_ gene: type A with multiple replicons and type B with IncI1 (Alpha) replicon. Type B plasmids exhibited superior adaptability and stability compared to type A plasmids within *Enterobacteriaceae*. Interestingly, although the type B (S808-p1) plasmid displayed the potential to spread to *Acinetobacter baumannii*, it failed to maintain stability in this species.

## 1. Introduction

*Salmonella* Enteritidis can contaminate eggs leading to asymptomatic infections in birds [1], which subsequently spread to humans, posing a significant threat to global health [2]. *Salmonella* primarily infects humans via the intestinal route and can manifest in a variety of diseases, with acute gastroenteritis being the most prevalent. This can result in either mild or fulminant diarrhea [3], often accompanied by low-grade fever, nausea, and vomiting. Presently, the *Salmonella* genus comprises two species: *S. bongori* and *S. enterica*. *S. enterica* is further categorized into six subspecies: *enterica* I, *salamae* II, *arizone* IIIa, *diarizone* IIIb, *houtenae* IV, and *indica* VI [4]. *S. enterica* I is predominantly responsible for human infections [5]. According to serotyping, *S. enterica* I can be further subdivided into *S.* Typhimurium, *S.* Paratyphi, *S.* Enteritidis, and so on. Among these, *S.* Enteritidis is a notable non-typhoid *Salmonella* strain capable of causing foodborne illnesses with a significant global impact on human health that cannot be overlooked [6].

Ceftriaxone, a vital antibiotic, is utilized in the treatment of *S.* Enteritidis infections. Resistance to ceftriaxone typically arises from the production of extended-spectrum β-lactamases (ESBLs), which possess the ability to hydrolyze expanded-spectrum cephalosporins, such as cefotaxime, ceftriaxone, ceftazidime, and cefepime, as well as monobactams like aztreonam [7]. Historically, the predominant families of ESBLs were the TEM and SHV enzymes. However, since the early 2000s, CTX-M enzymes have emerged as the most commonly encountered ESBLs [8]. CTX-M enzymes are classified as class A ESBLs and encompass multiple variants that currently dominate ESBL prevalence in clinical and community settings [9]. According to the annual surveillance data from the China Antimicrobial Surveillance Network (http://www.chinets.com/Data/AntibioticDrugFast, accessed on 14 March 2023 and 14 March 2024), 380 and 503 strains of *S.* Enteritidis exhibited resistance to ceftriaxone, accounting for 12.4% and 20.7% of resistance in 2022 and 2023, respectively. These data suggest a notable increase in ceftriaxone resistance within *S.* Enteritidis, a development that urgently calls for attention.

Plasmids serve as crucial vectors for the transmission of ESBLs, especially conjugable plasmids, which play a pivotal role in ESBL dissemination. Plasmids harboring ESBLs often contain numerous mobile genetic elements, such as IS*Ecp1*, IS*Cr1*, and IS*26*, which are associated with the transfer and transmission of ESBLs [10]. For instance, IS*26* facilitates the dissemination of antibiotic resistance genes (ARGs) through the conservative reaction and by forming translocatable units (TUs) [11]. Moreover, IS*26* can be inserted upstream of a resistance gene, creating hybrid promoters that enhance the expression of resistance genes. Previous studies have identified that IS*26* was involved in forming the promoter responsible for *bla*_BES-1_ expression as observed through sequence analysis of the IncP6-type plasmid (12,957 bp) in *Serratia marcescens* [12].

As a significant foodborne pathogen, *S.* Enteritidis contributes to ceftriaxone resistance. The emergence of ceftriaxone-resistant strains not only poses a threat to global public health and safety but also presents new therapeutic challenges. Therefore, understanding the related resistance mechanisms and transmission characteristics is paramount for public health. In this study, we elucidated the ceftriaxone resistance mechanism, plasmid characteristics, and plasmid transmission traits of ceftriaxone-resistant *S.* Enteritidis (CRO-RSE) isolated from Hangzhou First People’s Hospital, Zhejiang Province, from 2010 to 2020. We employed scientific research methods, including plasmid gene analysis, antibiotic sensitivity testing, conjugation experiments, growth curve analysis, and plasmid stability assessment. These findings provide a crucial research foundation for the surveillance and control of CRO-RSE.

## 2. Results

### 2.1. Clinical Information and Antimicrobial Susceptibility Testing

A total of 235 isolates of *S.* Enteritidis were obtained from Hangzhou First People’s Hospital, China, between 2010 and 2020. The majority of the strains were isolated from the Infectious Disease Department (79/235), with the remaining strains originating from the Intestinal Diseases Department (71/235), Pediatric Department (69/235), and other departments (16/235). The age distribution of these strains ranged from 0 to 88 years, predominantly from adults (117/235), followed by children (60/235), the elderly (40/235), and infants (18/235). Infants were defined as younger than 2 years old, children as 2–15 years old, adults as 16–60 years old, and the elderly as older than 60 years old. Isolates were obtained from various sources including stool (222/235), blood (11/235), and pus (2/235) samples. Of the total strains, 43.84% (101/235) were isolated from males and 56.16% (134/235) were isolated from females. Most of these isolates were obtained from patients with gastroenteritis, enteritis, or gastrointestinal disorders (187/235). Additionally, 50 strains (one from blood, and 49 from stool) originated from patients with symptoms of upper respiratory tract infection, 86% of which were from pediatric patients. Among the collected 235 *S.* Enteritidis strains, a total of 20 isolates exhibited resistance to ceftriaxone with a minimum inhibitory concentration (MIC) >256 µg/mL (8.51%, 20/235) from the Respiratory Medicine Department (1/20), Pediatric Department (9/20), and Intestinal Diseases Department (10/20). Furthermore, the resistance rates of 235 *S.* Enteritidis to other antimicrobial agents were as follows: ciprofloxacin 4.26% (10/235), azithromycin 19.15% (45/235), ampicillin 79.15% (186/235), cefoxitin 1.28% (3/235), imipenem 0, compound sulfamethoxazole 6.81% (16/235), chloramphenicol 5.11% (12/235), and tetracycline 28.51% (67/235).

### 2.2. Molecular Characteristics of Ceftriaxone-Resistant Salmonella Isolates

**Figure 1:**
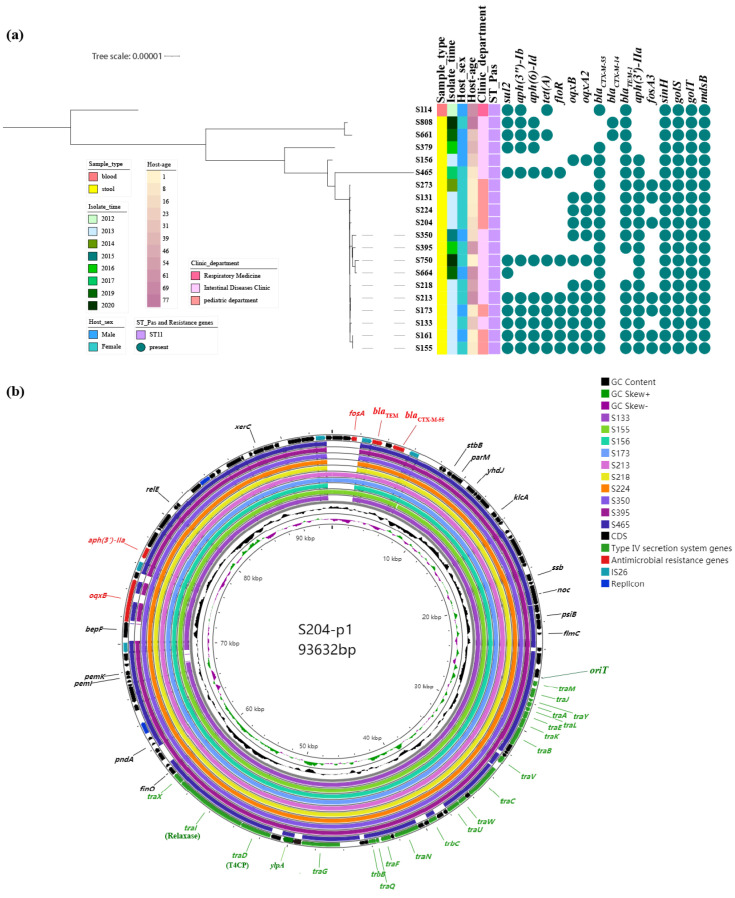
Molecular characteristics of CRO-RSE. (**a**) Core-genome phylogeny of 20 CTX-M-type-positive *S.* Enteritidis strains, based on the reference sequence of *S.* Enteritidis S114. The tree scale indicates the number of allelic differences. (**b**) Comparative analysis of ten CRO-RSE isolates, identified solely via short-read sequencing, carrying the *bla*_CTX-M-55_ gene, using S204-p1 as the reference sequence.

A total of 20 CRO-RSE isolates collected over the past decade underwent short-read sequencing, followed by long-read whole-genome sequencing of nine representative samples. Phylogenetic trees were constructed using the 20 CRO-RSE isolates, revealing that isolates harboring the same CTX-M-type enzymes clustered together, except for S114, which originated from a blood sample and formed a distinct cluster (Figure 1a and Figure S1). MLST analysis identified all 20 CRO-RSE isolates as belonging to ST11, characterized by the following MLST profile: *aroC 5*, *dnaN 2*, *hemD 3*, *hisD 7*, *purE 6*, *sucA 6*, *thrA 11*. Additionally, 18 strains carried *bla*_CTX-M-55_, while two strains carried *bla*_CTX-M-14_, both located on plasmids. IncX1, IncFII(pHN7A8), IncFIB(S), and IncFII(S) were associated with plasmids harboring *bla*_CTX-M-55_. Furthermore, among 18 strains carrying *bla*_CTX-M-55_, ARGs were located on plasmids (*fosA3*; *aph(3′)-IIa*; *bla*_TEM_; *aph(6)-Id*; *aph(3″)-Ib*; *sul2*; *oqxB*; *oqxA2*; *aph(6)-Id*; *tet(A)*; *floR*) (Table 1, Figure 1b).

### 2.3. Dissemination Characteristics of Ceftriaxone-Resistant Salmonella Isolates

#### 2.3.1. Analysis of Plasmid Structure Harboring bla_CTX-M-55_

Among the 18 CRO-RSE isolates harboring *bla*_CTX-M-55_, 17 shared a common genetic structure surrounding IS*26-bla*_TEM_*-hypothetical protein-bla*_CTX-M-55_-IS*26*, whereas S114 had a unique genetic structure: IS*Ecp1-bla*_CTX-M-55_*-hypothetical protein*. Additionally, plasmid S664 lacked some of the type IV secretion system (T4SS) genes (*traM*, *traJ*, *traY*, *traA*, *traL*, *traE*, *traK*, *traB*, *traV*, *trbC*, *traW*, *traU*, *traC*, *traN*, *traF*, *traQ*, *traB)* compared to other plasmids harboring *bla*_CTX-M-55_ (Figure 1b and Figure S2a). Concerning S114, it exhibited high similarity to pRHBSTW-00218_2 from *Enterobacter hormaechei* in wastewater influent in the UK (CP056650, coverage 100%, identity 99.99%), pKP4823_3 from *Klebsiella pneumoniae* in *Homo sapiens* in China (CP082793, coverage 100%, identity 99.98%), p20 from *Shigella sonnei* in *Homo sapiens* in Belgium (CP099778, coverage 98%, identity 99.80%), and pHNRD174 from *Escherichia coli* in ducks in China (KX246268, coverage 100%, identity 99.97%) (Figure S2b). All these plasmids belonged to the IncI1-type plasmid group, confirming the transmission of the *bla*_CTX-M-55_-bearing IncI1-type plasmid across different genera of bacteria and between the environment, animals, and humans.

#### 2.3.2. Analysis of Plasmid Structure Harboring bla_CTX-M-14_

The S808 AGRs were located on plasmids. S661 carrying *bla*_CTX-M-14_ matched to S808-p1 (Figure S3a). S114-p1 carrying *bla*_CTX-M-55_ exhibited a structure similar to that of S808-p1, which carries *bla*_CTX-M-14_. Furthermore, S808-p1 shared a similar structure with plasmids from the National Center for Biotechnology Information (NCBI), including the unnamed plasmid in *Salmonella enterica* from ground turkey in the USA (CP022064, coverage 85%, identity 99.30%), the plasmid p5848A2 identified in *E. coli* from the Netherlands (LR743516, coverage 85%, identity 99.31%), and the plasmid pESBL20160056 detected in *E. coli* found in Homo sapiens in Denmark (MH472638, coverage 85%, identity 99.30%) (Figure S3b). This suggests that both *bla*_CTX-M-55_ and *bla*_CTX-M-14_ are carried as passenger genes within IncI1-type plasmids during their propagation.

### 2.4. Plasmid Fusion Event of Two Plasmids

**Figure 2:**
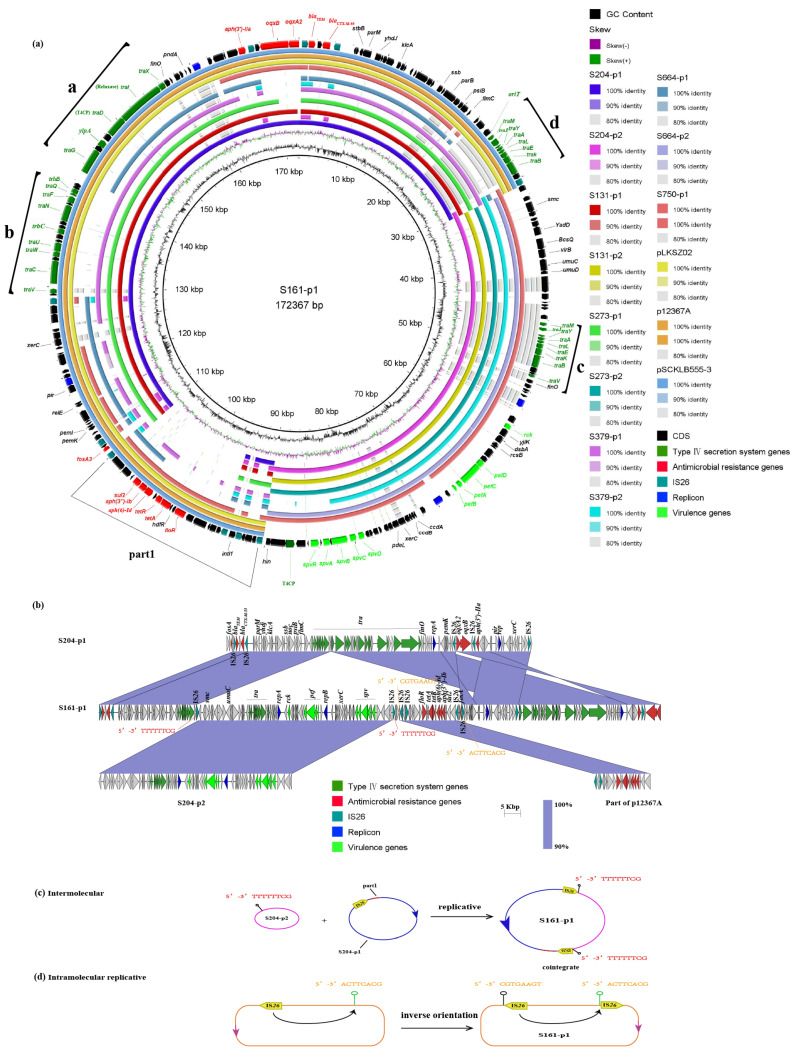
Plasmid fusion or cleavage event of two plasmids. (**a**) Fusion event observed at S204, S131, S273, S379, and S664 with reference to S161. The details of a–d parts are explanated in Table 2. (**b**) Detailed illustration of the fusion mechanism using S204 as an example. (**c**) Plots depicting the fusion patterns of the two plasmids. (**d**) Plots illustrating the pattern of intramolecular replicative transposition in S161-p1.

Plasmid fusion events were observed at sites S204, S131, S273, S379, and S664, with S161-p1 and S750-p1 serving as reference sequences. Among the fusion plasmids, S750-p1 lacked certain T4SS genes (*traV*, *trbC*, *traW*, *traU*, *traC*, *traN*, *traF*, *traQ*, *traB*, *traG*, *ylpA*, *traD*, *traI*, *traX*) compared to S161. Notably, S161-p1 has not been reported in the NCBI database, and the most similar plasmid is p12367A from *Salmonella enterica* (CP041177, coverage 68%, identity 99.99%). Using S204 as an example, S161-p1 consisted of S204-p1, S204-p2, and part1, which carried ARGs, including *floR*, *tet(A)*, *aph(6)-Id*, *aph(3″)-Ib*, *sul2*, and *fosA.* (Figure 2a). Part1 was identified in other bacterial plasmids, such as pLKSZ02 from *E. coli* (CP030283, coverage 100%, identity 100%), p12367A from *Salmonella enterica* (CP041177, coverage 100%, identity 99.99%), and pSCKLB555-3 from *K. pneumoniae* (CP043935, coverage 100%, identity 99.04%). Above all, this suggests that part1-carrying ARGs have been transmitted by plasmids among different bacterial genera (Figure 2a). For a more detailed analysis of the fusion mechanism, we found that a plasmid composed of S204-p2 (pSEV) that carried virulence genes (*rck*, *pefD*, *pefC*, *pefA*, *pefB*, *spvD*, *spvC*, *spvB*, *spvA*, *spvR*) and part1 was inserted into S204-p1. This process was mediated by IS*26* with 8 bp target site duplications (TSDs, TTTTTTCG) (Figure 2b,c). In addition, we observed intramolecular replicative transposition that led to the reversal of the segment between the original IS*26* and the targeted site. Evidence supporting this phenomenon was derived from the presence of 8 bp complementary sequences (Figure 2b,d).

### 2.5. Plasmid Type Classification in This Study

For subsequent plasmid studies, the plasmids detected by CRO-RSE were categorized into two types: type A and type B (93 kbp). Type A comprised subtypes A1 (130–170 kb) and A2 (93 kb). Subtype A1 plasmids, represented by S750-p1 and S161-p1, harbored *bla*_CTX-M-55_ and virulence genes, along with four replicons: IncX1, IncFII (pHN7A8), IncFIB (S), and IncFII (S). Subtype A1 plasmids contained four parts (parts a, b, c, and d) of the T4SS genes (Figure 2a). The differences between parts c and d included the absence of *traV* in part d and the truncation of *traJ* in part c. Additionally, S750-p1 lacked parts a and b of the T4SS compared to S161-p1. Furthermore, the analysis of plasmid fusion events revealed that the S204-p1 plasmid was derived from a portion of the S161-p1 plasmid. The subtype A2 plasmids, including S204-p1, S131-p1, S273-p1, S379-p1, and S664-p1, carried *bla*_CTX-M-55_ and two replicons, IncX1 and IncFII (pHN7A8). Subtype A2 plasmids contained three parts (parts a, b, and d) of the T4SS genes (Figure 2a). Additionally, S664-p1 lacked the b and d parts of the T4SS genes compared to the other subtype A2 plasmids. Type B plasmids, represented by S808-p1 and S114-p1, carried *bla*_CTX-M-55_ or *bla*_CTX-M-14_ and one replicon, IncI1 (Alpha). Type B plasmids contained part e of the T4SS genes (Figure S3b), which differed from parts a, b, c, and d. Both type A and B plasmids harbored the origins of transfer (oriT), relaxase, coupling protein (T4CP), and T4SS. Plasmids containing these four parts were considered conjugable plasmids.

### 2.6. Results of Plasmid Conjugation

The results of plasmid conjugation are shown in Table 2. Susceptibility testing of transconjugants revealed a significant increase in recipient resistance to ceftriaxone upon conjugation of plasmids containing the *bla*_CTX-M-type_ gene (Table 3). Subtype A2 plasmids were capable of conjugating with RrifSL1344, RrifJ53, and RrifATCC13883. Despite carrying oriT, relaxase, T4CP, and T4SS, S131-p1 failed to conjugate to any of the four recipients in our experiment. Furthermore, compared to other subtype A2 plasmids, S664-p1 could only conjugate to *E. coli* J53, potentially limiting its conjugative capability owing to the absence of a portion of the T4SS (absence of b and d). Similarly, compared to other subtype A1 plasmids, S750-p1 could only conjugate to *S.* typhi SL1344, likely because of the absence of a portion of the T4SS (absence of a and b). Additionally, S161 carrying a subtype A1 plasmid, S379 carrying a subtype A2 plasmid, and S808 carrying a type B plasmid were selected as donor bacteria, and RrifJ53 served as the recipient bacterium to calculate the conjugation frequency. The results revealed that the conjugation efficiencies of S161-p1, S379-p1, and S808-p1 were 1.65 × 10^−6^, 0.75 × 10^−4^, and 9.09 × 10^−4^, respectively. In summary, none of the subtype A1 plasmids were able to conjugate to *K. pneumoniae* ATCC13883, unlike the subtype A2 plasmids. Moreover, the conjugation efficiency of the subtype A2 plasmid was 45 times higher than that of the subtype A1 plasmid. Consequently, subtype A2 plasmids exhibited greater transmission potential than subtype A1 plasmids, implying that the absence of virulence genes and ARGs promoted plasmid dissemination. Regarding type B plasmids, both S114-p1 and S808-p1 were capable of conjugating with *S.* typhi SL1344, *E. coli* J53, and *K. pneumoniae* ATCC13883. Notably, the type B plasmid (S808-p1) could conjugate with *Acinetobacter baumannii* ATCC17978, indicating its potential to spread to *A. baumannii*. Furthermore, the conjugation efficiency of the type B plasmid was 12 times higher than that of the subtype A2 plasmid, indicating that the type B plasmid (S808-p1) had greater transmission capacity than the type A plasmid.

### 2.7. Fitness Cost Analysis of Different Plasmids under Different Strain Backgrounds

**Figure 3:**
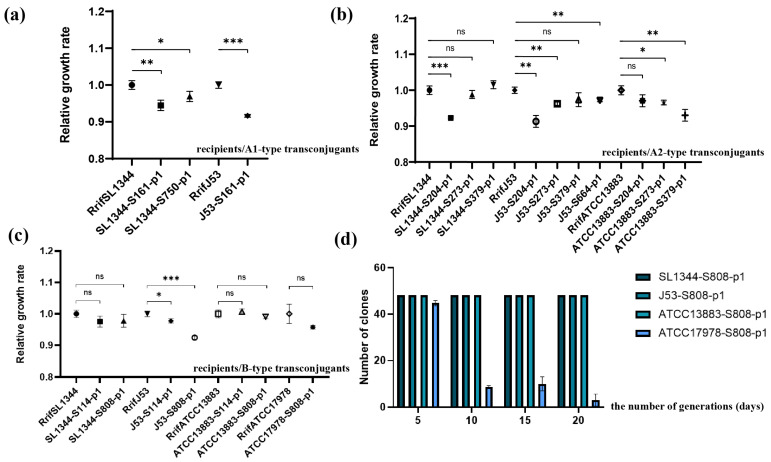
Comparison of relative growth rates of recipients carrying subtype A1 (**a**), A2 (**b**), and B-type (**c**) plasmids. (**d**) Plasmid stability experiments. The *x*-axis represents the number of generations of transconjugants, while the *y*-axis represents the number of transconjugants that still contain S808-p1. ns: *p* value ≥ 0.05, not significant; *: *p* value is from 0.01 to 0.05, significant; **: *p* value is from 0.001 to 0.01, very significant; ***: *p* value < 0.001, very significant.

Subtype A1 plasmids exhibited an increased fitness cost in various *Enterobacteriaceae* species (SL1344 and J53) (Figure 3a). Subtype A2 plasmids displayed varying fitness costs across different genera of *Enterobacteriaceae* (SL1344, J53, ATCC13883) (Figure 3b). Overall, within *Enterobacteriaceae*, certain transconjugants carrying the subtype A2 plasmids exhibited no fitness cost, whereas all those harboring the subtype A1 plasmids displayed an increased fitness cost. This suggests that the subtype A2 plasmids have better adaptability than the subtype A1 plasmid within *Enterobacteriaceae*. Additionally, type B plasmids increased the fitness costs only in *E. coli* J53 (Figure 3c). Consequently, the type B plasmids exhibit better adaptability than the type A plasmids.

### 2.8. Results of Plasmid Stability

Due to its higher transmissibility compared to type A plasmids, the type B plasmid (S808-p1) holds the potential to disseminate to *A. baumannii*. Transconjugants SL1344-S808-p1, J53-S808-p1, ATCC13883-S808-p1, and ATCC17978-S808-p1 were employed to assess the stability of type B plasmids within *Enterobacteriaceae* or *A. baumannii*. The S808-p1 plasmid exhibited stability in SL1344-S808-p1, J53-S808-p1, and ATCC13883-S808-p1 transconjugants over 20 consecutive days of passage. Conversely, strain ATCC17978-S808-p1 began losing the plasmid on day 5, with a plasmid loss rate of 6.94%. This loss rate stabilized at 81.94% on day 10 and 79.17% on day 15, ultimately reaching 93.75% by day 20 (Figure 3d). Despite the potential for the type B plasmid (S808-p1) to disseminate to *A. baumannii*, it could only maintain a stable presence for five days.

## 3. Discussion and Conclusions

In this study, we investigated 20 strains of CRO-RSE collected from Hangzhou First People’s Hospital, Zhejiang Province. Our findings revealed a close association between the spread of *bla*_CTX-M-type_ genes and the presence of IS*26* and IS*Ecp1* elements. Analysis of the plasmid structure indicated a highly conserved fundamental backbone, with regions carrying ARGs showing frequent alterations. These ARG-containing regions consistently harbored one or several mobile elements (eg. IS*26* and IS*Ecp1*), suggesting multiple insertion or recombination events due to mobile elements. Consequently, these regions could act as hotspots for homologous recombination [13]. A study reporting on *S. enterica* demonstrated that IS26-mediated composite transposon may facilitate the dissemination of *bla*_CTX-M-55_ [14]. Moreover, numerous studies on different serotypes of *S. enterica* also observed the transmission of *bla*_CTX-M-type_ genes in association with IS*26* and IS*Ecp1* [15,16,17,18]. Additionally, ceftriaxone-resistant isolates S161 and S750, collected in 2013 and 2020, respectively, exhibited a unique plasmid configuration where two plasmids harboring virulence genes and ARGs were fused, mediated by IS*26*. The emergence of such fusion plasmids, encoding both ARGs and virulence genes, presents a novel challenge to public health. Therefore, continuous monitoring of IS*26*-mediated transmission of significant pathogens is essential. In 2021, a study reported the presence of two hybrid plasmids mediated by IS*26* and Tn*6952* in *S.* Enteritidis [19]. However, this study did not assess the conjugative ability of plasmids through conjugation experiments.

A previous study in Guangdong, China, reported that the plasmids IncI1 and IncFII were likely the primary replicons contributing to the prevalence of *bla*_CTX-M-55_ in *E. coli* [20]. Additionally, previous reports indicated a close association between IncF-type plasmids and ESBL-producing strains of *E. coli* [21]. In our study, replicons IncI1(Alpha), IncFII(S), IncFII(pHN7A8), IncFIB(S), and IncX1 were associated with the spread of *bla*_CTX-M-55._ Several previous studies reported that the IncI1 plasmids carrying the *bla*_CTX-M-type_ genes conferred resistance to ceftriaxone in *Salmonella* [3,15,22]. In this study, we observed a similar trend. Importantly, we found, for the first time, that the IncI1 plasmid carrying the *bla*_CTX-M-type_ gene had the potential to spread across *Enterobacteriaceae* to *A. baumannii* through conjugation experiments. However, its stability in *A. baumannii* was limited, persisting for only approximately 5 days. While conjugation and growth curve experiments indicated that the Inc1 (Alpha)-type plasmid (type B plasmid) exhibited superior characteristics over type A plasmids in terms of transmissibility and fitness cost, it was noteworthy that the presence of type B plasmids (only three strains) was not prevalent among the 20 CRO-RSE samples collected from 235 *S.* Enteritidis. However, given the advantages of Inc1 (Alpha)-type plasmids in terms of transmission capacity and fitness cost, they are likely to emerge as primary vectors for the transmission of the *bla*_CTX-M-type_ gene in the future, warranting further investigation into their activities.

## 4. Materials and Methods

### 4.1. Collection of Isolates and Clinical Information

A total of 235 strains of *S.* Enteritidis were isolated from patients across various clinical departments of Hangzhou First People’s Hospital between 2010 and 2020. Colonies with correct characteristics, such as colorless transparency, with or without a black center, were selected from SS agar and streaked to ensure purity. Subsequently, the selected strains were amplified and resuspended in 20% glycerol broth before being stored in a −80 °C freezer for subsequent experiments. Serological agglutination tests were conducted using the Salmonella Diagnostic Serum Test Kit (Ningbo Tianrun Biopharmaceutical Company Limited, Ningbo, China) for the identification of *S.* Enteritidis. Clinical data, including collection date, gender, age, place of origin, department, and disease, were retrieved from the hospital’s network system, with identifiers concealed to ensure privacy.

### 4.2. Bacterial Isolate Identification and Antibiotic Susceptibility Test

All 235 isolates were identified using matrix-assisted laser desorption ionization–time of flight (MALDI-TOF) mass spectrometry systems (Bruker Daltonics, Bremen, Germany). Antibiotic susceptibility testing was conducted for all collected isolates, covering azithromycin, ampicillin, cefoxitin, imipenem, cotrimoxazole, carbenicillin, tetracycline, ceftriaxone, and ciprofloxacin, through the disk diffusion method. Additionally, the MICs of transconjugants and 20 CRO-RSE, which include ceftriaxone, were determined using the broth dilution method. Interpretation of the results was based on the 2023 Clinical and Laboratory Standards Institute guidelines (CLSI) [23] and the 2022 European Committee on Antimicrobial Susceptibility Testing [24].

### 4.3. Whole-Genome Sequencing of CRO-RSE Strains

We selected CRO-RSE for whole-genome sequencing, and genomic DNA was extracted using a QIAamp DNA Mini Kit (QIAGEN, Hilden, Germany). Whole-genome sequencing was performed on the Illumina HiSeq X-Ten platform (Illumina, San Diego, CA, USA) employing a 150 bp paired-end strategy. The reads were assembled using Shovill (https://github.com/tseemann/shovill, accessed on 15 May 2023) [25]. Then, nine CRO-RSE isolates, eight strains carrying *bla*_CTX-M-55_ and one strain carrying *bla*_CTX-M-14,_ were subjected to Nanopore MinION long-read sequencing (Oxford Nanopore Technologies, Oxford, UK). The filtered MinION raw reads were utilized and processed using Raven v1.1.10 [26], employing default parameters. Subsequently, Trycycler [27] was employed to validate the circularity of the complete genome sequence. Any errors were rectified using Illumina reads through Pilon v1.24 [28].

### 4.4. Genetic Structure Surrounding bla_CTX-M-type_ Gene

For ceftriaxone-resistant isolates, ARGs were identified by ABRicate v0.8.13 (https://github.com/tseemann/abricate, accessed on 23 July 2023) based on the ResFinder database (http://genomicepidemiology.org/, accessed on 23 July 2023) [29]. Multilocus sequence typing (MLST) was determined using the MLST 2.0 server, and the Inc-type plasmid of the strain was screened using plasmidfinder [30]. Additionally, nine CRO-RSE isolates were annotated by Prokka [31], and then Easyfig v2.2.5 (http://easyfig.sourceforge.net/, accessed on 5 August 2023) [32], proksee (https://proksee.ca/, accessed on 8 August 2023) [33], and BRIG v0.95 (https://sourceforge.net/projects/brig/, accessed on 10 August 2023) [34] were used to align different plasmids. In addition, we also used NCBI BLAST (https://blast.ncbi.nlm.nih.gov/Blast.cgi, accessed on 13 August 2023) to seek similar plasmids containing *bla*_CTX-M-55_ or *bla*_CTX-M-14_.

### 4.5. Phylogenetic Analysis

A total of 20 CRO-RSE strains were utilized for phylogenetic analysis based on their SNPs. Genome alignment was established using Snippy (https://github.com/tseemann/snippy, accessed on 10 June 2023) with default parameters [35]. The unrooted maximum-likelihood phylogenetic tree was constructed employing FastTree [36]. The tree was then visualized and annotated using the iTOL tree [37].

### 4.6. Plasmid Transfer Assays

Nine strains (S204, S131, S273, S379, S664, S161, S750, S114, and S808) served as donor bacteria, which underwent short-read and long-read sequencing. Recipients comprised RrifSL1344, RrifJ53, RrifATCC13883, and RrifATCC17978. Donor plasmids carrying the *bla*_CTX-M-type_ gene were transferred to the recipients. Donors and recipients were cultivated in 2 mL of Mueller Hinton Broth (MHB) for four hours at 37 °C on a shaker. Subsequently, 100 μL of both donors and the recipients were mixed, and 20 µL of the mixture was spotted onto a 0.22 μm membrane (Merck, Darmstadt, Germany) affixed to Mueller Hinton Agar (MHA), followed by overnight incubation at 37 °C. The next day, the bacteria on the membrane were dissolved in MHB, and 20 μL was spotted onto a double antibiotic plate, which was then incubated overnight at 37 °C. Transconjugants were selected on a double antibiotic plate containing rifampicin (1000 mg/L) and ceftriaxone (64 mg/L). Antibiotic sensitivity testing results were interpreted according to CLSI guidelines (2023) [11]. The presence of *bla*_CTX-M-type_ genes, replicon genes, and *Salmonella’s* housekeeping gene *aroC* in transconjugants was confirmed via PCR. Transconjugants were also identified using MALDI-TOF. Conjugation frequency was calculated as the number of transconjugants per donor.

### 4.7. Bacterial Growth Curve Assay

Three single colonies of both recipients and transconjugants were selected and inoculated into MHB, with blank controls set up simultaneously. Ten microliters of bacterial solution were added to 1 mL of MHB culture medium, equivalent to a 1:100 dilution of the overnight bacterial culture. This diluted solution was then added into a honeycomb plate, with 200 µL per well, conducting three technical replicates for each sample while simultaneously establishing a blank control. The honeycomb plate was then placed in an automatic growth curve instrument (Growth Curves, Turku, Finland). The parameters were set to 37 °C, and the optical density at 600 nm (OD600) was measured every 5 min for a total of 20 h. The growth rate during the logarithmic phase was calculated, and the relative growth rate of the strain was determined using R script [38], with the recipients serving as the reference.

### 4.8. Plasmid Stability Assay

After overnight culture, the bacterial solution was diluted 1:1000 with 2 mL of MHB medium and incubated overnight with shaking at 200 rpm. This process was repeated daily up to the 20th generation. During this time, bacterial solutions from the 5th, 10th, 15th, and 20th generations were diluted onto MHA plates, and 48 single colonies were streaked onto both an antibiotic plate containing ceftriaxone (64 mg/L) and an MHA plate to verify ceftriaxone-resistant plasmids. Transconjugants that grew on both ceftriaxone plates and MHA were considered positive for plasmid retention, while those that could not grow on ceftriaxone plates but grew on MHA were considered negative for plasmid retention. The plasmid loss rate was calculated using the following formula: Plasmid loss rate = (mean number of negative clones from three replicates/48) × 100.

## Figures and Tables

**Table 1:** ^#^ Genomic characteristics of nine CRO-RSE isolates.

	* Location ^&^ (Plasmid_Type)	Size (bp)	GC (%)	*bla*_CTX-M-type_	Other Resistance Genes	Plasmid Replicons
S273	Chromosome	4,679,990	52.18	-	*sinH*; *golS*; *golT*; *mdsB*	-
	p1 (A2)	90,399	51.75	*bla*_CTX-M-55_	*fosA3*; *aph(3′)-IIa*; *bla*_TEM_	IncX1; IncFII(pHN7A8)
	p2 (pSEV)	59,372	51.95	-	-	IncFIB(S); IncFII(S)
S379	Chromosome	4,679,476	52.18	-	*sinH*; *golS*; *golT*; *mdsB*	-
	p1 (A2)	113,301	49.97	*bla*_CTX-M-55_	*bla*_TEM_; *aph(6)-Id*; *aph(3″)-Ib*; *sul2*	IncX1; IncFII(pHN7A8); IncN
	p2 (pSEV)	57,128	51.85	-	*bla*_TEM_	IncFIB(S); IncFII(S)
	p3	3904	51.13	-	-	Col156
S131	Chromosome	4,680,029	52.18	-	*sinH*; *golS*; *golT*; *mdsB*	-
	p1 (A2)	93,985	52.01	*bla*_CTX-M-55_	*fosA3*; *bla*_TEM_; *aph(3′)-IIa*; *oqxB*; *oqxA2*	IncX1; IncFII(pHN7A8)
	p2 (pSEV)	59,372	51.95	-	-	IncFIB(S); IncFII(S)
S204	Chromosome	4,679,989	52.18	-	*sinH*; *golS*; *golT*; *mdsB*	-
	p1 (A2)	93,632	52.02	*bla*_CTX-M-55_	*oqxA2*; *oqxB*; *aph(3′)-IIa*; *fosA3*; *bla*_TEM_	IncX1; IncFII(pHN7A8)
	p2 (pSEV)	59,372	51.95	-	-	IncFIB(S); IncFII(S)
	p3	52,891	43.83	-	-	IncFII(p96A); IncFII(S)
S161	Chromosome	4,679,615	52.18	-	*sinH*; *golS*; *golT*; *mdsB*	-
	p1 (A1)	172,367	52.65	*bla*_CTX-M-55_	*bla*_TEM_; *oqxA2*; *oqxB*; *aph(3′)-IIa*; *fosA3*; *sul2*; *aph(3″)-Ib*; *aph(6)-Id*; *tet(A)*; *floR*	IncX1; IncFII(pHN7A8); IncFIB(S); IncFII(S)
S114	Chromosome	4,679,992	52.18	-	*sinH*; *golS*; *golT*; *mdsB*	-
	p1 (B)	86,191	49.66	*bla*_CTX-M-55_	-	IncI1(Alpha)
	p2 (pSEV)	64,327	51.76	-	*bla*_TEM_	IncFIB(S); IncFII(S)
	p3	29,336	47.22	-	*bla*_TEM_; *aph(6)-Id*; *aph(3″)-Ib*; *tet(A)*; *aph(6)-Id*; *aph(3″)-Ib*; *sul2*	IncX1
	p4	6647	48.52	-	-	ColRNAI
S808	Chromosome	4,679,741	52.18	-	*sinH*; *golS*; *golT*; *mdsB*	-
	p1 (B)	93,230	49.88	*bla*_CTX-M-14_	-	IncI1(Alpha)
	p2 (pSEV)	64,327	51.76	-	*bla*_TEM_	IncFIB(S); IncFII(S)
	p3	24,484	44.75	-	*bla*_TEM_; *aph(6)-Id*; *aph(3″)-Ib*; *sul2*	IncX1
S664	Chromosome	4,679,298	52.18	-	*sinH*; *golS*; *golT*; *mdsB*	-
	p1 (A2)	67,207	52.28	*bla*_CTX-M-55_	a*ph(3′)-IIa*; *sul2*; *bla*_TEM_	IncFII(pHN7A8); IncX1
	p2 (pSEV)	59,323	51.95	-		IncFIB(S); IncFII(S)
S750	Chromosome	4,680,774	52.18	-	*sinH*; *golS*; *golT*; *mdsB*	
	p1 (A1)	116,054	53.10	*bla*_CTX-M-55_	*bla*_TEM_; *oqxA2*; *oqxB*; *aph(3′)-IIa*; *aph(6)-Id*; *aph(3″)-Ib*; *sul2*; *tet(A)*; *floR*;	IncFII(S); IncX1

^#^ Genomic characteristics of nine CRO-RSE isolates: The genomic characteristics of the 9 CRO-RSE isolates sequenced by both short-read and long-read sequencing methods. * Location: p1-p4 refer to plasmid 1-plasmid 4. ^&^ (Plasmid_type): In this study, plasmids carrying *bla*_CTX-M-type_ gene were divided into A1, A2, and B-type (2.5 below for details). *S.* Enteritidis usually harbors a virulence plasmid (pSEV) (sequence ID: NZ_JACEGM010000032.1, 58,347 bp) that is similar to S808-p2, S114-p2, S204-p2, S131-p2, S273-p2, and S664-p2 (coverage: 99–100%, identity: 99.99–100%). pSEV was also similar to S379-p2 (coverage: 86%, identity: 100%).

**Table 2:** Plasmid transfer results.

Isolate Strain	The Plasmid Transferring	T4SS	*bla*_CTX-M_	Plasmid_Type	Recipient SL1344	Recipient J53	Recipient ATCC 13883	Recipient ATCC 17978
S273	S273-p1	a, b, d	55	A2	+	+	+	–
S379	S379-p1	a, b, d	55	A2	+	+	+	–
S131	S131-p1	a, b, d	55	A2	–	–	–	–
S204	S204-p1	a, b, d	55	A2	+	+	+	–
S664	S664-p1	a	55	A2	–	+	–	–
S161	S161-p1	a, b, c, d	55	A1	+	+	–	–
S750	S750-p1	c, d	55	A1	+	–	–	–
S114	S114-p1	e	55	B	+	+	+	–
S808	S808-p1	e	14	B	+	+	+	+

“+”: referring to successfully conjugated strains. “–”: referring to unsuccessfully conjugated strains. Part a: *traG*, *ylpA*, *traD* (T4CP), *traI* (Relaxase), *traX*. Part b: *traV*, *trbC*, *traW*, *traU, traC*, *traN*, *traF*, *traQ*, *traB*. Part c: *traM*, *traJ*, *traY*, *traA*, *traL*, *traE*, *traK*, *traB*, *traV*. Part d: *traM*, Δ*traJ*, *traY*, *traA*, *traL*, *traE*, *traK*, *traB*. Part e: Relaxase, *trbC*(T4CP), *trbB*, *trbA*, *traY*, *traX*, *traW*, *traV*, *traU*, *traT*, *traS*, *traR*, *traQ*, *traP*, *traO*, *traN*, *traM*, *traL*, *traK*, *traJ*, *traI*, *traH*. (T4SS can be seen in Figure 2 and Figure S3).

**Table 3:** Results of ceftriaxone susceptibility testing in the transconjugants.

Recipient (CRO_MIC)	S161-p1	S114-p1	S204-p1	S808-p1	S750-p1	S273-p1	S379-p1	S664-p1
RrifSL1344 (0.125)	1024	2048	2048	512	512	2048	1024	-
RrifJ53 (0.06)	2048	1024	1024	512	-	1024	512	512
Rrif13883 (0.06)	-	2048	2048	512	-	1024	1024	-
Rrif17978 (8)	-	-	-	>2048	-	-	-	-

The first column in parentheses represents the MIC of ceftriaxone for the recipient, while the ceftriaxone MICs of the donors were all higher than 256 µg/mL. The rows correspond to the recipients, with the columns indicating the conjugated plasmids, and the numbers denote the MIC of ceftriaxone for the transconjugants (µg/mL). The recipients included rifampicin-induced *S.* Typhimurium SL1344 (RrifSL1344), *E. coli* J53 (RrifJ53), *K. pneumoniae* ATCC13883 (RrifATCC13883), and *A. baumannii* ATCC17978 (RrifATCC17978).

## Data Availability

The datasets presented in this study can be found in online repositories (https://www.ncbi.nlm.nih.gov/, accessed on 4 November 2023) and the accession number(s) can be found in BioProject: PRJNA1023753.

## Supplementary Materials

The following supporting information can be downloaded at: https://www.mdpi.com/article/10.3390/antibiotics13050456/s1, Figure S1. Visualization of the number of single nucleotide polymorphism (SNP) variant sites among 20 CRO-RSE. Figure S2. Analysis of plasmids harboring *bla*_CTX-M-55_. (a) Comparative analysis of plasmids harboring *bla*_CTX-M-55_ with the reference sequence S204-p1. (b) Linear comparison of *bla*_CTX-M-55_-bearing plasmids from the National Center for Biotechnology Information (NCBI) database with the reference sequence S114-p1. Figure S3. Analysis of plasmids harboring *bla*_CTX-M-14_. (a) Comparative analysis of the short read sequencing data from S661 with the reference sequence S808-p1. (b) Linear comparison of plasmids structurally similar to S808-p1.
