# Supplementary material for: Dissemination of Ceftriaxone-Resistant Salmonella Enteritidis Harboring Plasmids Encoding blaCTX-M-55 or blaCTX-M-14 Gene in China
Source: Antibiotics (Basel). 2024 May 16;13(5):456. doi: 10.3390/antibiotics13050456 (PMC11117602; doi:10.3390/antibiotics13050456)
Supplement: Supplementary file 1 [file antibiotics-13-00456-s001.zip › antibiotics-2970310-supplementary.pdf]

antibiotics

MDPI

Supplementary Materials

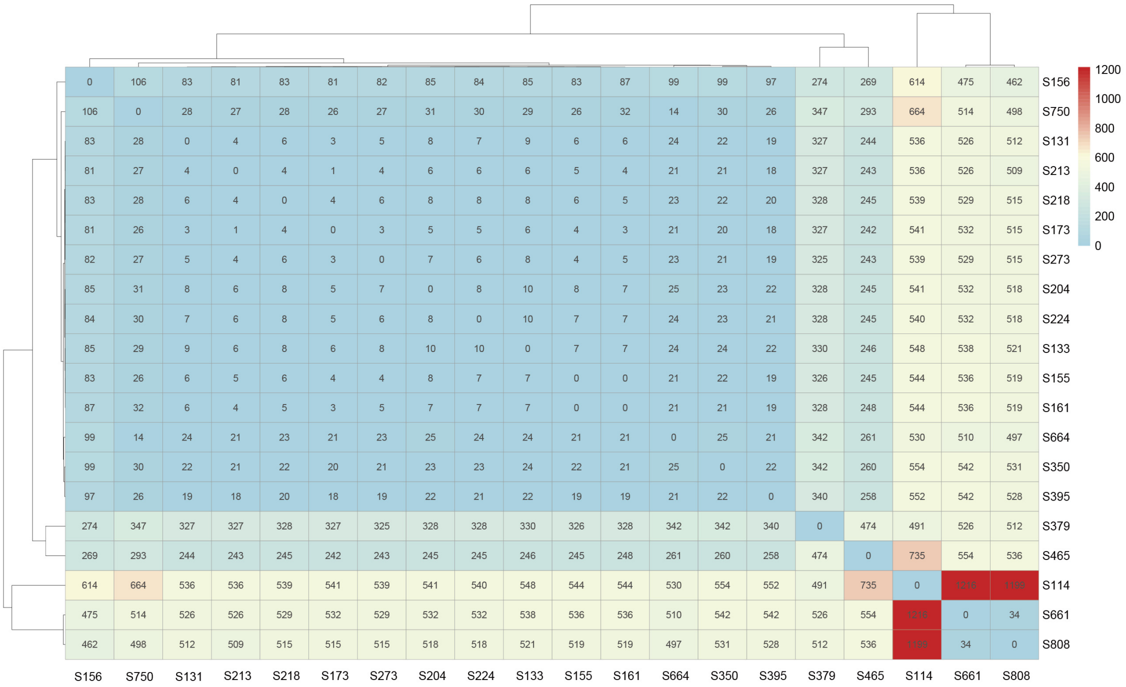

Figure S1. Visualization of the number of single nucleotide polymorphism (SNP) variant sites among 20 CRO-RSE.

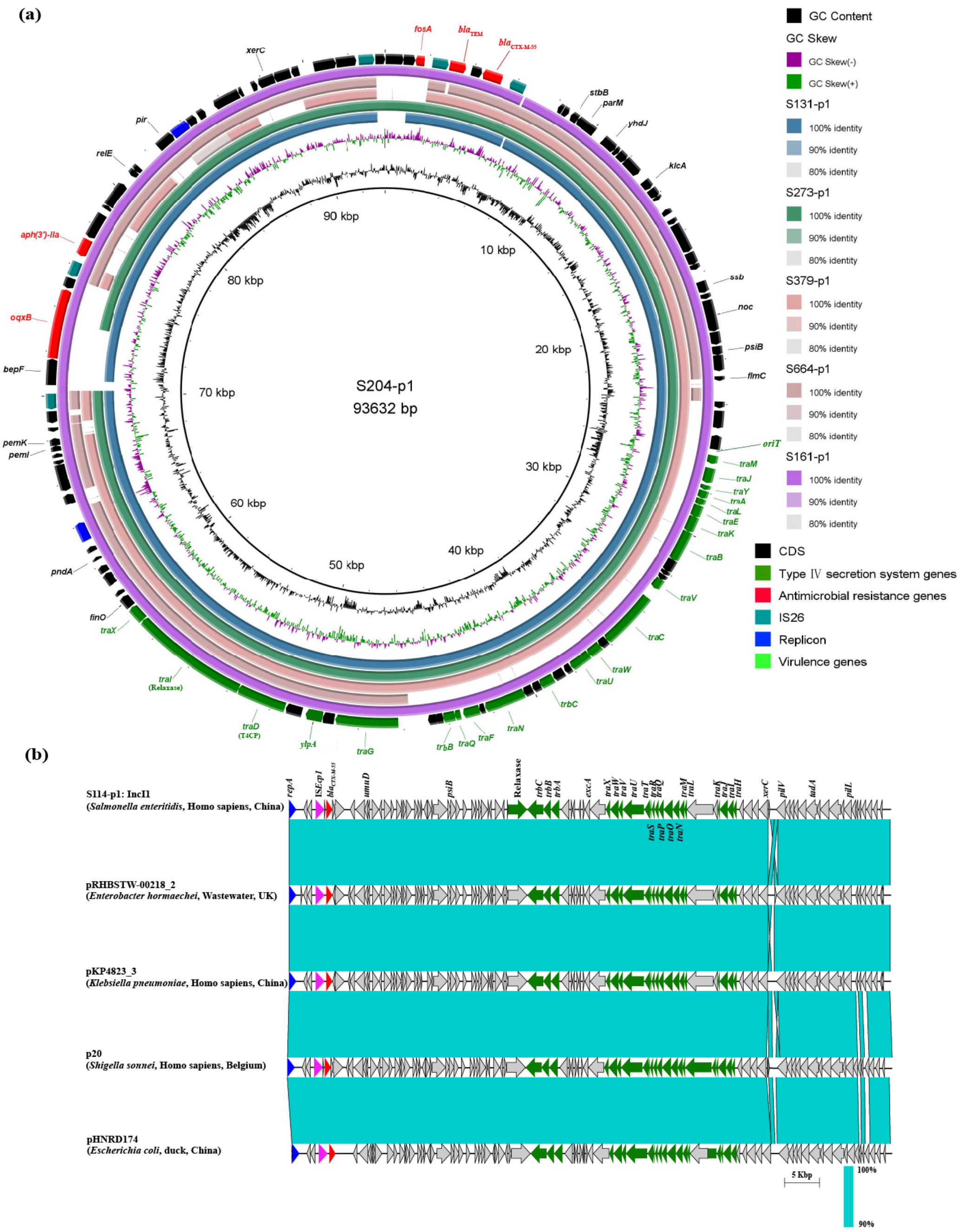

Figure S2. Analysis of plasmids harboring *bla*$_{CTX-M-55}$. (**a**) Comparative analysis of plasmids harboring *bla*$_{CTX-M-55}$ with the reference sequence S204-p1. (**b**) Linear comparison of *bla*$_{CTX-M-55}$-bearing plasmids from the National Center for Biotechnology Information (NCBI) database with the reference sequence S114-p1.

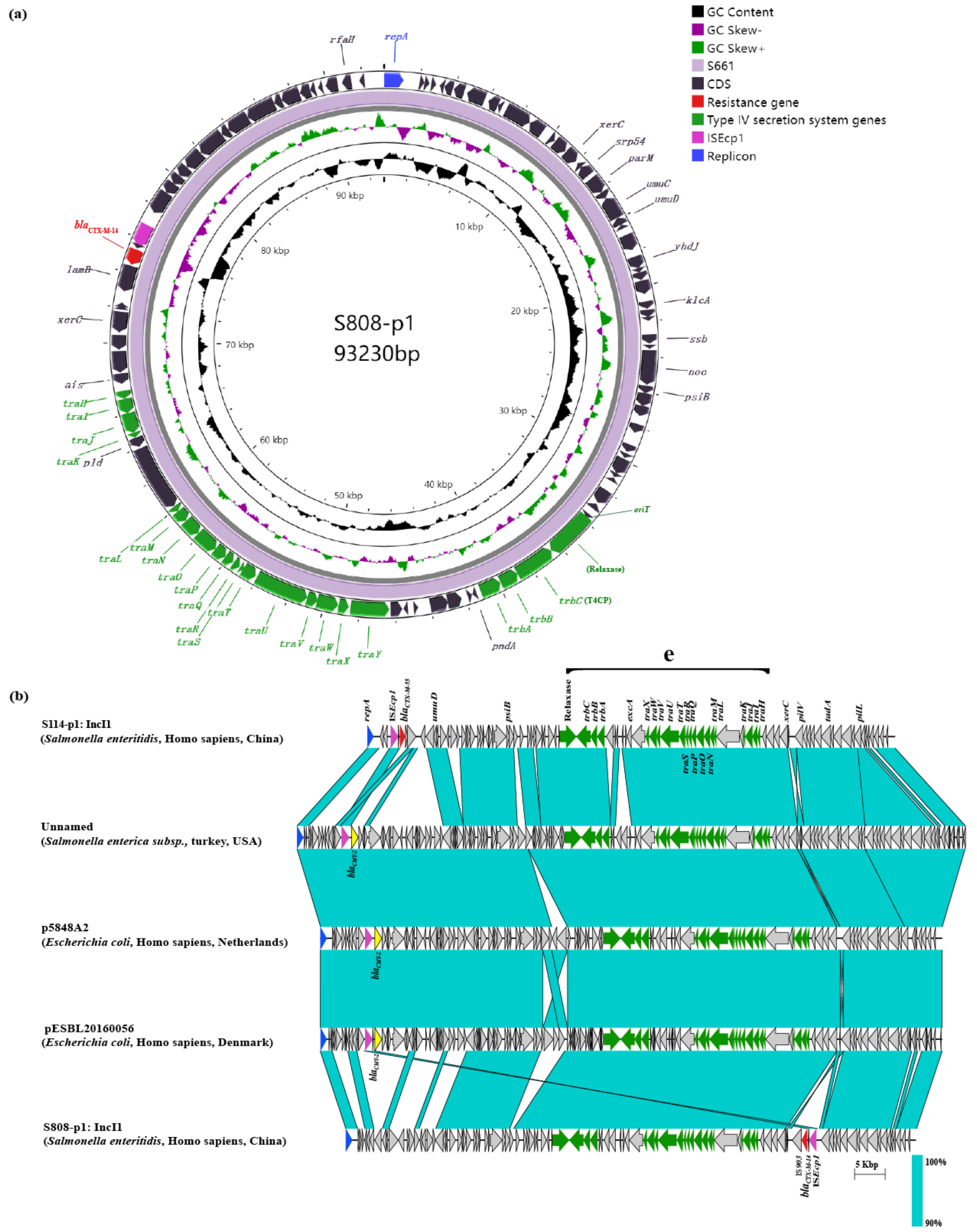

Figure S3. Analysis of plasmids harboring *bla*CTX-M-14. (**a**) Comparative analysis of the short read sequencing data from S661 with the reference sequence S808-p1. (**b**) Linear comparison of plasmids structurally similar to S808-p1.
